# Cigarette smoke-induced dysbiosis: comparative analysis of lung and intestinal microbiomes in COPD mice and patients

**DOI:** 10.1186/s12931-024-02836-9

**Published:** 2024-05-10

**Authors:** Vincent Laiman, Hsiao-Chi Chuang, Yu-Chun Lo, Tzu-Hsuen Yuan, You-Yin Chen, Didik Setyo Heriyanto, Fara Silvia Yuliani, Kian Fan Chung, Jer-Hwa Chang

**Affiliations:** 1 Department of Radiology, Faculty of Medicine, Public Health, and Nursing, Universitas Gadjah Mada – Dr. Sardjito Hospital, Yogyakarta, Indonesia; 2 School of Respiratory Therapy, College of Medicine, Taipei Medical University, 250 Wuxing Street, Taipei, 11031 Taiwan; 3 Division of Pulmonary Medicine, Department of Internal Medicine, Shuang Ho Hospital, Taipei Medical University, New Taipei City, Taiwan; 4 Cell Physiology and Molecular Image Research Center, Wan Fang Hospital, Taipei Medical University, Taipei, Taiwan; 5 National Heart and Lung Institute, Imperial College London, London, UK; 6 The Ph.D. Program for Neural Regenerative Medicine, College of Medical Science and Technology, Taipei Medical University, Taipei, Taiwan; 7 Department of Health and Welfare, College of City Management, University of Taipei, Taipei, Taiwan; 8 Industrial Ph.D. Program of Biomedical Science and Engineering, National Yang Ming Chiao Tung University, Taipei, Taiwan; 9 Department of Biomedical Engineering, National Yang Ming Chiao Tung University, Taipei, Taiwan; 10 Department of Anatomical Pathology, Faculty of Medicine, Public Health, and Nursing, Universitas Gadjah Mada – Dr. Sardjito Hospital, Yogyakarta, Indonesia; 11 Department of Pharmacology and Therapy, Faculty of Medicine, Public Health, and Nursing, Universitas Gadjah Mada, Yogyakarta, Indonesia; 12 Division of Pulmonary Medicine, Departments of Internal Medicine, Wan Fang Hospital, Taipei Medical University, Taipei, Taiwan; 13 Present Address: Collaboration Research Center for Precision Oncology based Omics- PKR Promics, Universitas Gadjah Mada, Yogyakarta, Indonesia

**Keywords:** Cigarette smoke, Emphysema, Inflammation, Intestine, Lung, Microbiome

## Abstract

**Background:**

The impact of cigarette smoke (CS) on lung diseases and the role of microbiome dysbiosis in chronic obstructive pulmonary disease (COPD) have been previously reported; however, the relationships remain unclear.

**Methods:**

Our research examined the effects of 20-week cigarette smoke (CS) exposure on the lung and intestinal microbiomes in C57BL/6JNarl mice, alongside a comparison with COPD patients’ intestinal microbiome data from a public dataset.

**Results:**

The study found that CS exposure significantly decreased forced vital capacity (FVC), thickened airway walls, and induced emphysema. Increased lung damage was observed along with higher lung keratinocyte chemoattractant (KC) levels by CS exposure. Lung microbiome analysis revealed a rise in Actinobacteriota, while intestinal microbiome showed significant diversity changes, indicating dysbiosis. Principal coordinate analysis highlighted distinct intestinal microbiome compositions between control and CS-exposed groups. In the intestinal microbiome, notable decreases in Patescibacteria, Campilobacterota, Defferibacterota, Actinobacteriota, and Desulfobacterota were observed. We also identified correlations between lung function and dysbiosis in both lung and intestinal microbiomes. Lung interleukins, interferon-ɣ, KC, and 8-isoprostane levels were linked to lung microbiome dysbiosis. Notably, dysbiosis patterns in CS-exposed mice were similar to those in COPD patients, particularly of Global Initiative for Chronic Obstructive Lung Disease (GOLD) stage 4 patients. This suggests a systemic impact of CS exposure.

**Conclusion:**

In summary, CS exposure induces significant dysbiosis in lung and intestinal microbiomes, correlating with lung function decline and injury. These results align with changes in COPD patients, underscoring the important role of microbiome in smoke-related lung diseases.

**Supplementary Information:**

The online version contains supplementary material available at 10.1186/s12931-024-02836-9.

## Introduction

Cigarette smoke (CS) stands as a leading cause of preventable deaths worldwide [1]. While the prevalence of smoking is declining in developed countries, it is still on the rise in many developing countries, particularly in Asia [2]. Approximately half of smokers will develop serious smoking-related diseases, including chronic obstructive pulmonary disease (COPD), cardiovascular disease, and various cancers [1, 3]. In lung disease such as COPD, chronic airway inflammation is a key patho-mechanism, often attributed to persistent acute inflammation caused by CS inhalation [1]. Despite numerous studies reporting the impact of CS on lung diseases, the mechanisms by which CS modulates changes resulting in lung disease remain unclear.

The lung microbiome, encompassing viable and nonviable microbiota residing in parenchymal tissues, plays important role in preserving lung health and may be altered in pulmonary diseases [4, 5]. For instance, a previous study involving COPD subjects revealed that dysbiosis of the lung microbiome is associated with COPD severity, exacerbations, and adverse clinical outcomes [6]. This dysbiosis, characterized by the outgrowth of pathogenic bacteria, is also linked to immune system impairment observed in COPD [7]. Furthermore, an imbalance in the lung microbiome has been associated with inflammation, pathological changes in the airways, immune reactions, and the worsening of clinical symptoms in COPD [8–10]. Additionally, both active smoking and being subjected to secondhand smoke are associated with the presence of potentially pathogenic bacteria [11]. Another study demonstrated that CS exposure in mice for 72 days disrupts intestinal microbiome homeostasis and promotes systemic inflammation [12]. Considering smoking as a significant risk factor for COPD development, this suggests that CS exposure can lead to microbiome dysbiosis and potentially contribute to COPD development. Nonetheless, alterations in both the lung and intestinal microbiomes exert substantial negative effects on human health.

The exact links between lung and intestinal microbiomes, lung function, and injury due to CS induced COPD remain unclear. The objective of this study was to explore how CS exposure affects lung function, phenotypic lung changes, and the lung and intestinal microbiomes in mice, drawing parallels with COPD.

## Materials and methods

### Animals

Six-week-old male C57BL/6JNarl mice were procured from the National Laboratory Animal Center in Taipei, Taiwan. They were accommodated in a controlled environment with a constant temperature of 22 ± 2 °C, relative humidity (RH) of 55% ± 10%, and a 12:12-hour light-dark cycle. All animal experiments adhered to the regulations set by the animal and ethics review committee of the Laboratory Animal Center at Taipei Medical University (Taipei, Taiwan; IACUC: LAC-2021-0260).

### CS-induced emphysema model

Figure S1 in Supplementary Information (SI) shows the schematic experimental design of the study. The Mice were constantly exposed to CS using a CS generation system following equipped with a whole-body exposure system (CS group). At the same time, a control group of mice was exposed to high-efficiency particulate air (HEPA)-filtered air devoid of cigarette smoke. The CS generation system and the whole-body exposure system (TECNIPLAST, Italy) have been previously reported [13]. The CS group mice were exposed to the CS for 20 weeks (4 cigarettes/hour, 8 h/day, and 5 days/week) [14], whereas the control group mice were exposed to CS-free air for 20 weeks simultaneously. At week 21, fecal samples were collected in sterile condition and lung function tests were performed followed by necropsy Zoletil 50 (Virbac; Taipei, Taiwan) (0.1 mL/mouse) and Rompun 2% (Bayer Korea, Ltd.; Ansan, Gyeonggi-do, Korea) (0.05 mL/mouse) via intraperitoneal injection before collection of the serum, bronchoalveolar lavage fluid (BALF), lung and intestinal samples in sterile condition as previously described [13]. Briefly, blood collection was performed via the submandibular method. After collection, the blood samples were kept at room temperature for one hour before being centrifuged at 200G under 4 °C for 10 min. The resulting serum was separated and stored at -80ºC. The BALF was obtained by rinsing the lungs three times with 0.5 ml of sterile phosphate-buffered saline (PBS). The BALF samples underwent centrifugation at 200G for 10 min at 4 °C, and the resulting supernatant was collected and stored at -80ºC for future use. The exposure system is further detailed in Section S1 in SI.

### Lung function measurement

The pulmonary function was measured by Flexivent (SCIREQ; Sterling, VA, US). Calibration was performed before the experiment in accordance with the manufacturer’s manual. The mice were inserted with the 24-gauge soft catheter and connected with the ventilator in Flexivent under 2–3% of isoflurane. The forced expiratory volume at 0.1 s (FEV0.1), forced vital capacity (FVC), peak expiratory flow (PEF), FEV0.1/FVC, inspiratory capacity, airway resistance and tissue damping were measured in this study.

### Airway wall thickness and mean linear intercept (MLI)

Lung samples were inflated with 10% neutral-buffered formalin by intratracheal instillation at a pressure of 25 cmH_2_O for 10 min. The lung tissues were then embedded in paraffin and cut into sections for staining with haematoxylin and eosin (H&E). Lung H&E images were obtained by Motic Easyscan Pro and Motic DSAssistant software (Motic, Xiamen, Fujian, China). The thickness of the airway wall was assessed through the use of ImageJ software (National Institute of Health, Bethesda, MD, USA) based on images captured at a 20x magnification. The MLI assessed the length of lines crossed and the number of grid lines intercepts in alveolar space from 10 non-overlapping fields of each lung sample according to previously described method (Crowley et al., 2019).

### Lung damage assessment

Lung damage assessment was performed by utilizing K-means clustering algorithm using ImageJ software. This algorithm sorted and ranked points on H&E slides based on staining intensity and density, indicative of varying injury severity [15, 16]. All pixels in tissue images were categorized into four clusters, including a background component and three clusters highly correlated with injured regions. To prevent potential bias in the relative clustering of heterogeneously injured tissue, each image of a single lobe was defined as a comparison reference and clustered together. Severe zones, characterized by maximal damage (red), Mild zones with active morphological remodeling (green), and Normal zones displaying homeostatic appearances (blue) were identified.

### Intestinal inflammation score

Intestines were fixed in 10% neutral-buffered formalin and embedded with paraffin. Intestinal tissue sections were then stained with H&E by routine methods. The inflammation score on distal colon sections was scored from 0 (minimal) to 4 (marked inflammation) according to a scoring scheme from a previous publication [17].

### Extraction of lung and intestinal tissue protein

Extraction of proteins in lung and intestinal tissue were performed by homogenizing tissues in buffer containing cell lysis reagent CelLytic MT (Sigma, St. Louis, MO, USA), 1% protease inhibitor, and 1% ethylenediaminetetraacetic acid (EDTA) by grinding the sample. Samples were centrifuged at 16,000 g in 4ºC centrifuge for 30 min. Supernatants were then collected and were stored at -80 °C for later use.

### Enzyme-linked immunosorbent assay

Enzyme-linked immunosorbent assay (ELISA) was performed to assess the interleukin (IL)-1β, IL-10, IL-17 A, interferon (IFN)-ɣ, chemokine (CXC motif) ligand 1/keratinocyte chemoattractant (CXCL1/KC) (R&D Systems, MN, USA), and 8-isoprostane (Cayman Chemicals, MI, USA) levels in BALF, serum, lung and intestine according to the manufacturers’ instructions. The BCA Protein Assay Reagent Kit (Bio-Rad, Hercules, CA, USA) was used to determine the proteins concentrations from the lysates. The concentrations of these markers measured in lung and intestinal tissues were normalized relative to the total protein content in lung and intestinal lysates, respectively.

### Microbiome DNA extraction and analysis

The DNA extraction and analysis of the lung and intestinal microbiome have been previously reported [18]. Briefly, the lobe of the lung was harvested under sterile conditions, and 10 mg fresh sample was used in QIAamp DNeasy Blood & Tissue Kits (Qiagen, Hilden, Germany) to extract DNA of lung bacteria of each mouse. For intestinal microbiome, we collected a minimum of two fecal samples using sterilized microtubes, promptly storing them at -80°C. The individual fecal samples from each mouse were combined, resulting in 220 mg of the mixed sample for subsequent DNA extraction. The amplification of the V3-V4 region of the 16S ribosomal (r)DNA gene was carried out using specific primers containing Illumina sequencing adapters and sample-specific barcodes. Subsequently, the amplified DNA was sequenced on an Illumina MiSeq sequencer. The universal primers 341F (5’-CCTACGGGNGGCWGCAG-3’) and 805R (5’-GACTACHVGGGTATCTAATCC-3’) with Illumina overhang adapter sequences in the forward (5’-TCGTCGGCAGCGTCAGATGTGTATAAGAGACAG-3’) and reverse (5’-GTCTCGTGGGCTCGGAGATGTGTATAAGAGACAG-3’) primers were utilized to target the V3-V4 highly variable region of the 16 S rDNA gene sequence. Detailed procedures for DNA extraction, 16 S rDNA gene amplification, sequencing, and analysis methods are provided in section S2 in the SI.

### Microbiome Data in COPD with Global Initiative for Chronic Obstructive Lung Disease (GOLD) classification patients

To see if the changes in the microbiome in CS-exposed mice were similar to those in humans with COPD, we retrieved publicly available 16 S rDNA microbiome datasets of COPD subjects from open access repositories for comparison. The Mendeley Data general repository (https://data.mendeley.com) was used to acquire pertinent processed datasets available to the public. Subsequently, this list was narrowed down by applying the following constraints: (1) Only lung or intestinal-related samples from COPD patients that were consistently post-processed were included and (2) each study should contain both samples from COPD subjects and from control subjects. This search resulted in one study [10]. The microbiome data from our dataset and the publicly available dataset were then normalized relative to the respective control samples. Comparative analysis was performed to identify the changes in microbiome between our CS-exposed mice dataset and the publicly available COPD dataset.

### Statistical analysis

We tested for a normal distribution with the Shapiro-Wilk’s test. For data with non-normal distribution, such as FEV, PEF, FEV0.1/FVC, inspiratory capacity, tissue damping, IL-17 A and 8-isoprostane in BALF, IL-17 A and KC in serum, and 8-isoprostane level in intestine, a non-parametric test using the Wilcoxon rank-sum test was used. Otherwise, student’s t-test was performed for comparisons of the groups. Chi-square test was employed to examine the difference in the inflammatory score. Alpha diversity metrics were computed utilizing the estimated richness function available in the phyloseq package. For beta diversity, unweighted unique fraction (UniFrac) principal coordinate analysis (PCoA) was conducted. Spearman’s correlation coefficients were performed to assess correlations: (1) between lung functions and the lung and intestinal microbiomes, (2) lung inflammation and the lung microbiomes, (3) intestinal inflammation and the intestinal microbiomes, and (4) between the lung and intestinal microbiomes. Visual representation of Spearman’s correlation was generated using RStudio (vers. 4.1.1) for macOS. Statistical analyses were carried out using GraphPad vers. 9 for macOS, with the significance level set at *p* < 0.05.

## Results

### CS exposure decreased lung function with increased airway wall thickness and emphysema development

**Fig. 1:**
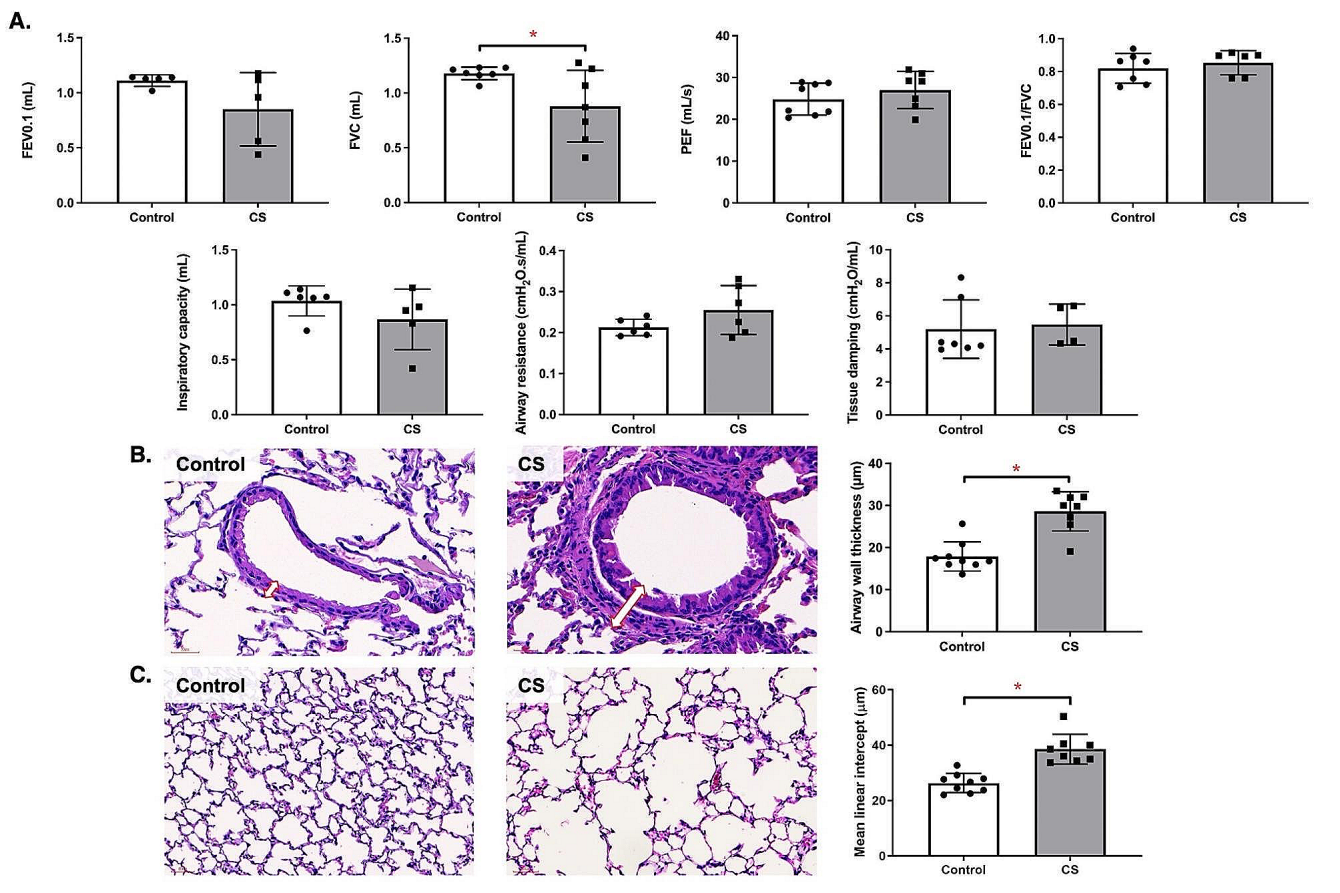
Lung function and structural changes after cigarette smoke (CS) exposure. **(A)** Lung function examination including the forced expiratory volume at 0.1 s (FEV0.1), forced vital capacity (FVC), peak expiratory flow (PEF), FEV0.1/FVC, inspiratory capacity, airway resistance and tissue damping (*n* = 6–8). **(B)** Representative histological images of airway wall thickness examination between control and CS-exposed mice. Arrow indicates thickness of airway wall (*n* = 8–9) (scale bar = 30 μm). **(C)** Representative histological images of the alveolar region with the alveolar sizes quantified by mean linear intercept (*n* = 8–9) (scale bar = 60 μm). ^*^p < 0.05

We observed that CS exposure decreased FVC (*p* < 0.05) (Fig. 1A). However, no significant difference was observed in FEV0.1, PEF, FEV0.1/FVC, inspiratory capacity, airway resistance, or tissue damping. The airway wall thickness observed in CS-exposed mice was significantly increased compared to control (*p* < 0.05) (Fig. 1B). The MLI scoring for lung alveolar sizes exhibited significant increase in the CS exposure group compared to the control group (*p* < 0.05) (Fig. 1C).

### CS exposure increased systemic inflammation

Figure S2 showed the inflammatory cytokines in BALF and serum. We did not see significant changes in the inflammatory cytokines in BALF after CS exposure (Figure S2A). However, we observed that CS exposure significantly increased IL-1β, IL-10, IFN-ɣ, and 8-isoprostane levels in the serum compared to control (*p <* 0.05) (Figure S2B).

### CS exposure increased lung severity and inflammation

**Fig. 2:**
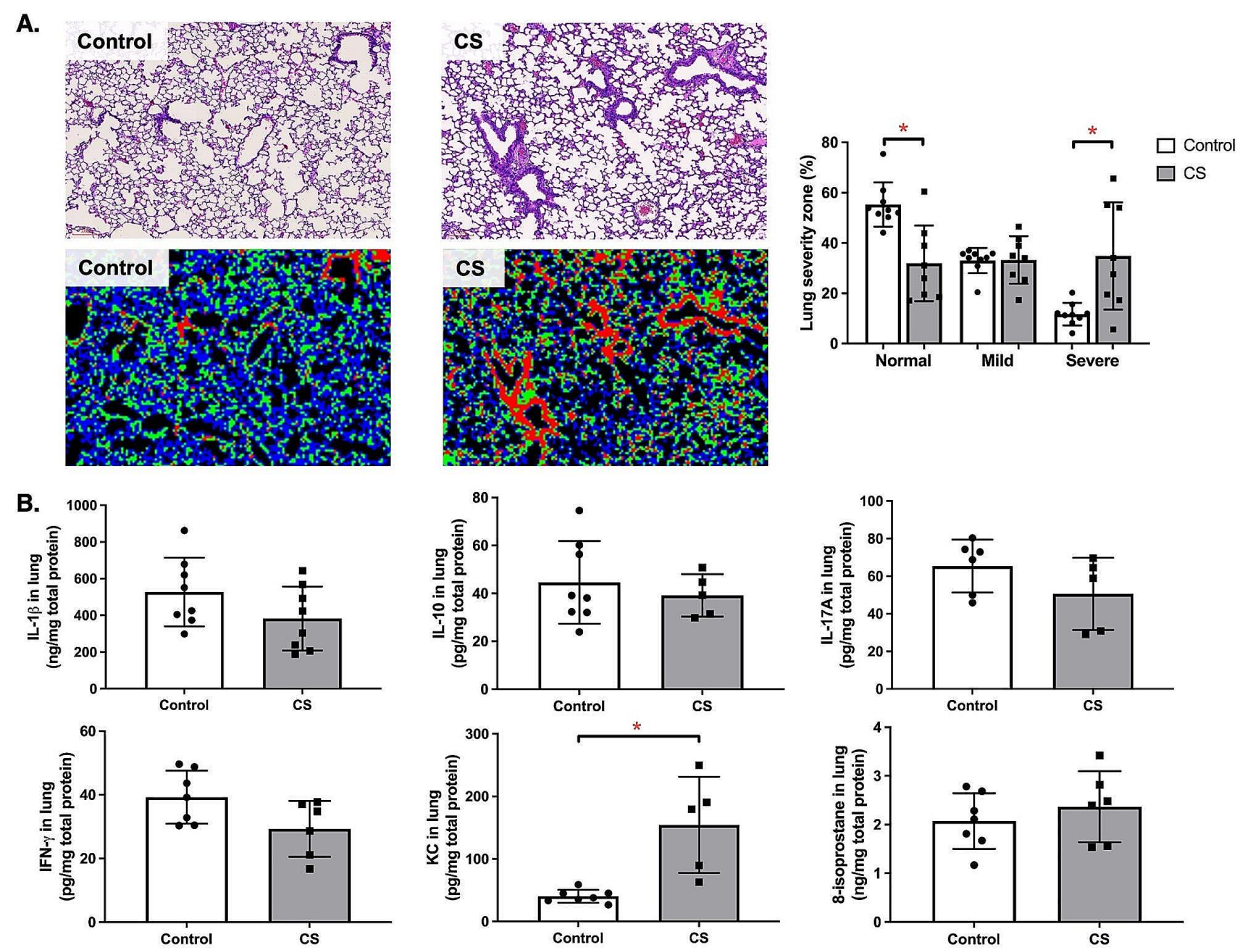
Lung damage and inflammation after cigarette smoke (CS) exposure. **(A)** Representative histological and K-means-clustered images of the alveolar region. The regions displaying homeostatic appearances (blue) were categorized as Normal zones, areas with active morphological remodeling (green) were labeled as Mild zones, and regions of maximal damage (red) were designated as Severe zones (*n* = 8–9) (scale bar = 100 μm). **(B)** Lung inflammatory markers including interleukin (IL)-1β, IL-10, IL-17 A, interferon (IFN)-ɣ, keratinocyte chemoattractant (KC), and 8-isoprostane in lung tissue normalized by total protein (*n* = 5–8). ^*^p < 0.05

Figure 2A shows the distinct zones of injury in the lung. We observed that CS exposure significantly decreased the normal zone% and increased the severe zone% in the lung (*p* < 0.05). Next, we observed that CS exposure significantly increased the KC in the lung compared to control group (*p* < 0.05) (Fig. 2B). However, no significant difference was observed in IL-1β, IL-10, IL-17 A, IFN-ɣ, or 8-isoprostane in the lung between control and CS exposure groups.

### CS exposure increased inflammatory responses in intestine

**Fig. 3:**
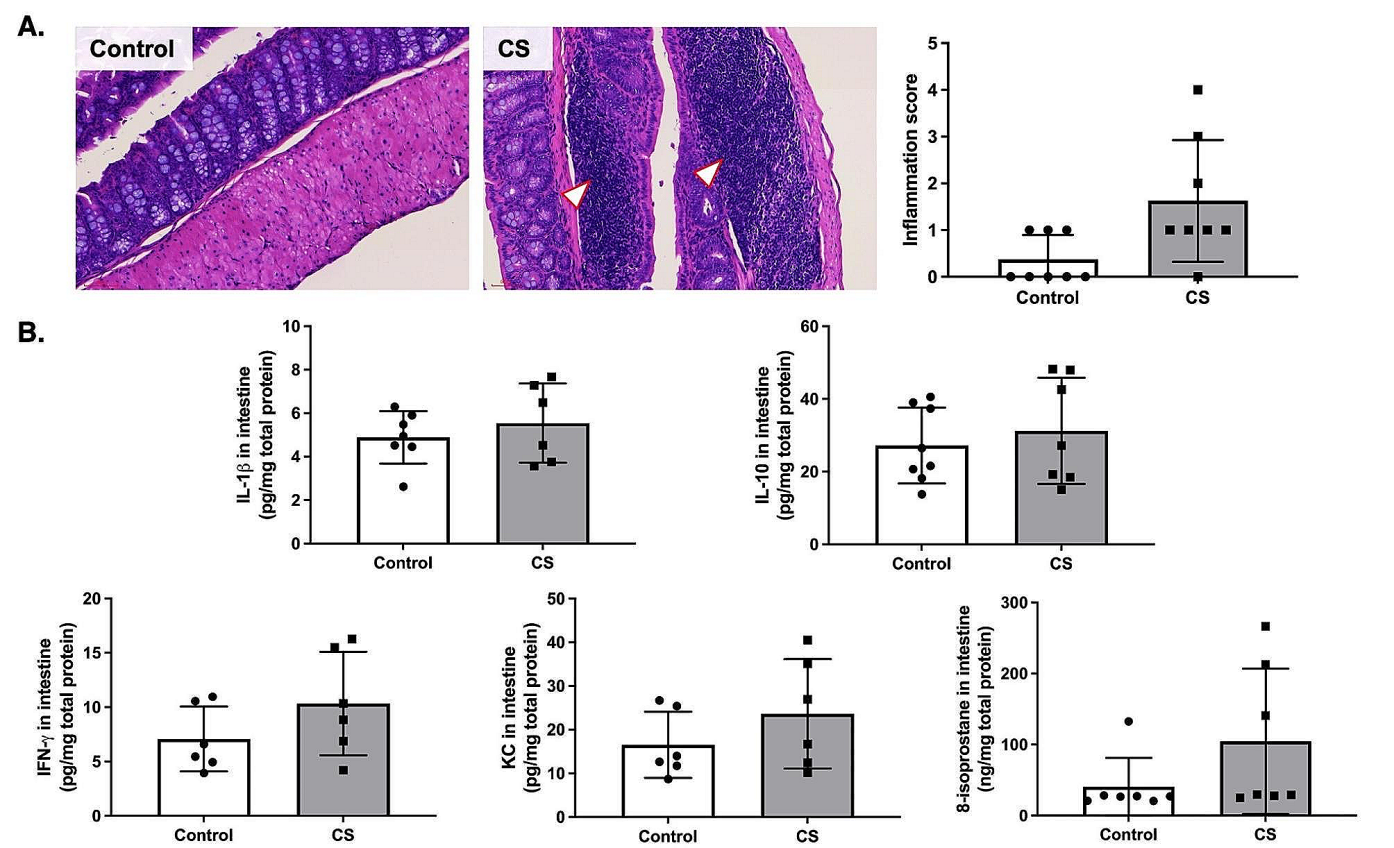
Intestinal inflammation after cigarette smoke (CS) exposure. **(A)** Representative histological images of the intestine. Arrowheads indicate inflammatory cells (*n* = 8) (scale bar = 60 μm). **(B)** Intestinal inflammatory markers including interleukin (IL)-1β, IL-10, interferon (IFN)-ɣ, keratinocyte chemoattractant (KC), and 8-isoprostane in intestinal tissue normalized by total protein (*n* = 6–8)

We observed increased inflammatory cell infiltrates in the intestines of CS-exposed mice (Fig. 3A). However, no significant difference was observed in the intestinal inflammatory score and inflammatory cytokines between groups (Fig. 3B).

### CS exposure altered bacterial compositions in lung

**Fig. 4:**
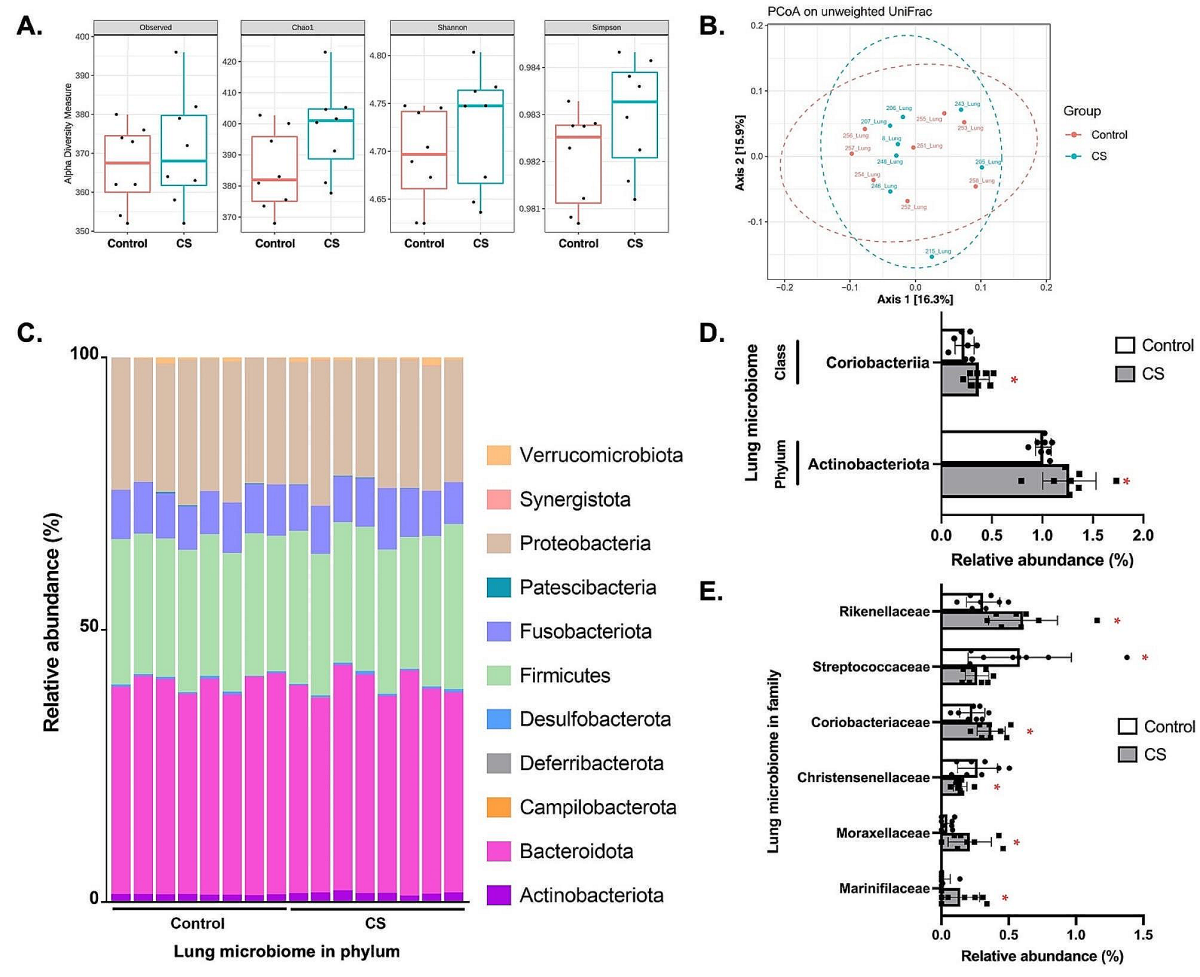
Lung microbiome analysis after cigarette smoke (CS) exposure. (**A**) Alpha diversity analysis including Observed, Chao1, Shannon, and Simpson indexes. (**B)** Beta diversity analysis of lung microbiome. **(C)** Composition of lung microbiome at the phylum level. (**D)** Comparisons of significantly different lung microbiome at the phylum and class level. (**E)** Comparisons of significantly different lung microbiome at the family level (*n* = 8). ^*^p < 0.05

As shown in Fig. 4A and B, both the alpha and beta diversity were not significantly different between CS exposure and control groups. In phylum level, 11 phyla were identified with Bacteroidota, Firmicutes, and Proteobacteria being the most predominant phylum (Fig. 4C). CS exposure significantly increased the Actinobacteriota in the phylum level and Coriobacteriia in the class level (*p* < 0.05) (Fig. 4D). Further analysis showed that in family level, CS exposure significantly increased Rikenellaceae, Coriobacteriaceae, Moraxellaceae, and Marinicellaceae relative abundance while decreasing Streptococcaceae and Christensellaceae relative abundance (*p* < 0.05) (Fig. 4E).

### CS exposure altered intestinal bacterial diversity and compositions

**Fig. 5:**
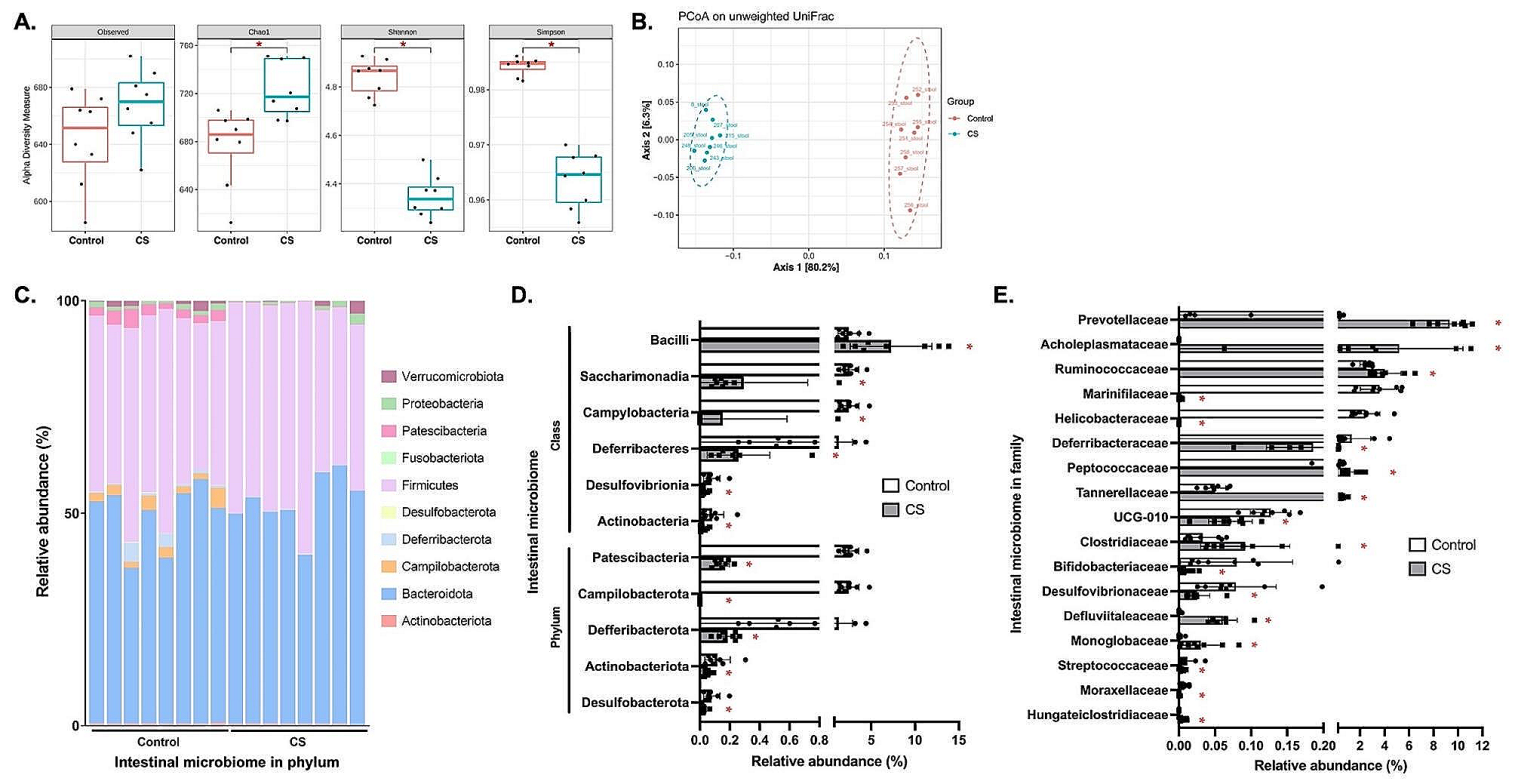
Intestinal microbiome analysis after cigarette smoke (CS) exposure. (**A**) Alpha diversity analysis including Observed, Chao1, Shannon, and Simpson indexes. (**B)** Beta diversity analysis of intestinal microbiome. **(C)** Composition of intestinal microbiome at the phylum level. (**D)** Comparisons of significantly different intestinal microbiome at the phylum and class level. (**E)** Comparisons of significantly different intestinal microbiome at the family level (*n* = 8). ^*^p < 0.05

Figure 5A and B show the alpha diversity and beta diversity in intestinal microbiomes between control and CS exposure groups. The microbiome richness (Chao1) was significantly increased by CS exposure (*p* < 0.05). The alpha diversity (Shannon) and evenness (Simpson) observed in CS group were significantly decreased compared to the control group (*p* < 0.05). The PCoA based on the unweighted UniFrac distance showed significant compositional differences between control and CS groups, with 80.2% and 6.3% variation shown by PC1 and PC2 principal components, respectively (*p* < 0.05). In phylum level, 10 phyla were found, with the Bacteroidota and Firmicutes being the predominant phylum in intestinal microbiome (Fig. 5C). CS exposure significantly decreased Patescibacteria, Campilobacterota, Defferibacterota, Actinobacteriota, and Desulfobacterota in phylum level (*p* < 0.05) (Fig. 5D). Of note, the Campilobacterota was not detected after CS exposure. In the class level, the relative abundance of Bacilli observed in CS group was significantly increased while the Saccharimonadia, Campylobacteria, Defferibacteres, Desulfovibrionia, and Actinobacteria were significantly decreased compared to control group (*p* < 0.05). Further analysis showed that CS exposure significantly altered the relative abundance of several microbiome in family level, with the most increase observed in Acholeplasmataceae and Prevotellaceae and most decrease observed in Marinifilaceae and Helicobacteraceae compared to control (*p* < 0.05) (Fig. 5E). Of note, the Acholeplasmataceae and Hungateiclostridiaceae were observed only after CS exposure whereas the Helicobacteraceae was not found after CS exposure.

### Correlation of lung functions with both lung and intestinal microbiome

**Fig. 6:**
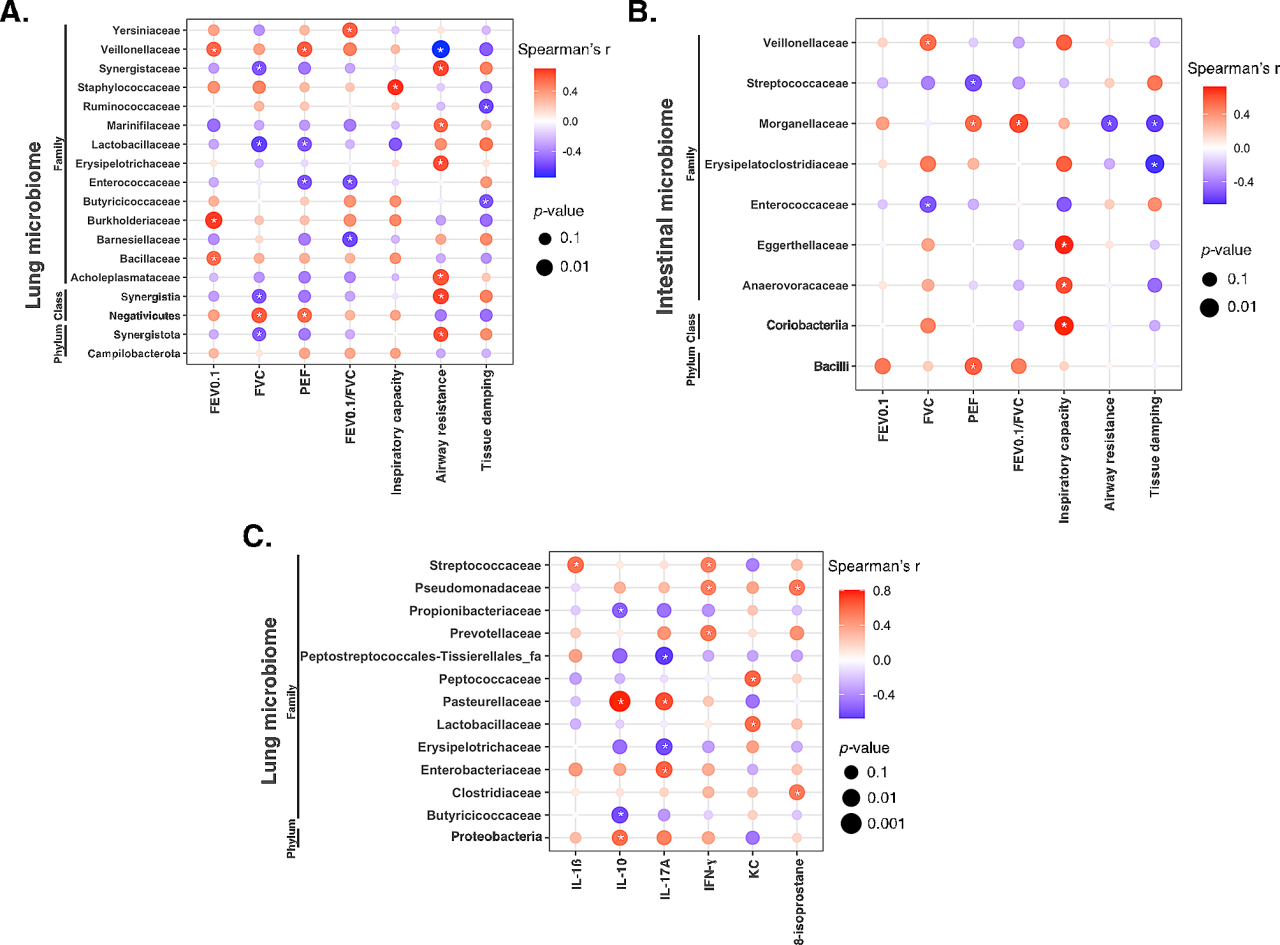
Heatmaps depicting correlation analyses. **(A)** Heatmap illustrating correlations between lung functions and the lung microbiome. **(B)** Heatmap displaying correlations between lung functions and the intestinal microbiome. **(C)** Heatmap showcasing correlations between the lung microbiome and lung inflammatory markers. The intensity of color reflects the correlation coefficient strength (red: positive correlation; blue: negative correlation). The size of the data points reflects the significance of the correlation. * *p* < 0.05

Figure 6A shows the correlation between lung microbiome in phylum, class, and family level with the lung functions. The FEV0.1 was positively correlated with Basillaceae, Burkholderiaceae, and Veillonellaceae (*p* < 0.05). The FVC was positively correlated with Negativicutes while negatively correlated with Synergistota, Synergistia, Lactobacillaceae, and Synergistaceae (*p* < 0.05). Additionally, the PEF was positively correlated with Negativicutes and Veillonellaceae while negatively correlated with Enterococcaceae and Lactobacillaceae (*p* < 0.05). The FEV0.1/FVC was positively correlated with Yernisiaceae while negatively correlated with Barnesiellaceae and Enterococcaceae (*p* < 0.05). Lung inspiratory capacity correlated positively with Staphylococcaceae (*p* < 0.05). Airway resistance was positively correlated with Synergistota, Synergistia, Acholeplasmataceae, Erysipelotrichaceae, Marinifilaceae, and Synergistaceae while negatively correlated with Veillonellaceae (*p* < 0.05). The lung tissue damping was negatively correlated with Butyricicoccaceae and Ruminococcaceae (*p* < 0.05).

The correlation between lung functions and intestinal microbiome is shown in Fig. 6B. FVC correlated positively with Veillonellaceae and negatively with Enterococcaceae (*p* < 0.05). PEF correlated positively with Bacilli and Morganellaceae, while negatively with Streptococcaceae (*p* < 0.05). FEV0.1/FVC was positively correlated with Morganellaceae, while lung inspiratory capacity correlated positively with Coriobacteriia, Anaerovoracaceae, and Eggerthelaceae (*p* < 0.05). Airway resistance negatively correlated with Morganellaceae, and lung tissue damping showed negative correlation with Erysipelatoclostridiaceae and Morganellaceae (*p* < 0.05).

### Correlation between lung inflammation and lung microbiome

We observed that the IL-1β in lung tissue correlated positively with Streptococcaceae (*p* < 0.05) (Fig. 6C). The IL-10 correlated positively with Proteobacteria and Pasteurellaceae while negatively with Butyricicoccaceae and Propionibacteriaceae (*p* < 0.05). The IL-17 A was positively correlated with Enterobacteriaceae and Pasteurellaceae while negatively correlated with Erysipelotrichaceae and Peptostreptococcales-Tissierelalles (*p* < 0.05). Positive correlation was observed between IFN-ɣ and Prevotellaceae, Pseuodomonadaceae, and Streptococcaceae (*p* < 0.05). The KC correlated positively with Lactobacillaceae and Peptococcaceae while 8-isoprostane correlated positively with Clostridiaceae and Pseudomonadaceae (*p* < 0.05).

### Correlation between intestinal inflammation and intestinal microbiome

The intestinal microbiome showed several strong correlations with intestinal inflammation including IL-1β, IL-10, IFN-ɣ, and KC (Figure S3). The detailed results were presented in Section S2.1 in SI.

### Correlation between lung microbiome and intestinal microbiome

**Fig. 7:**
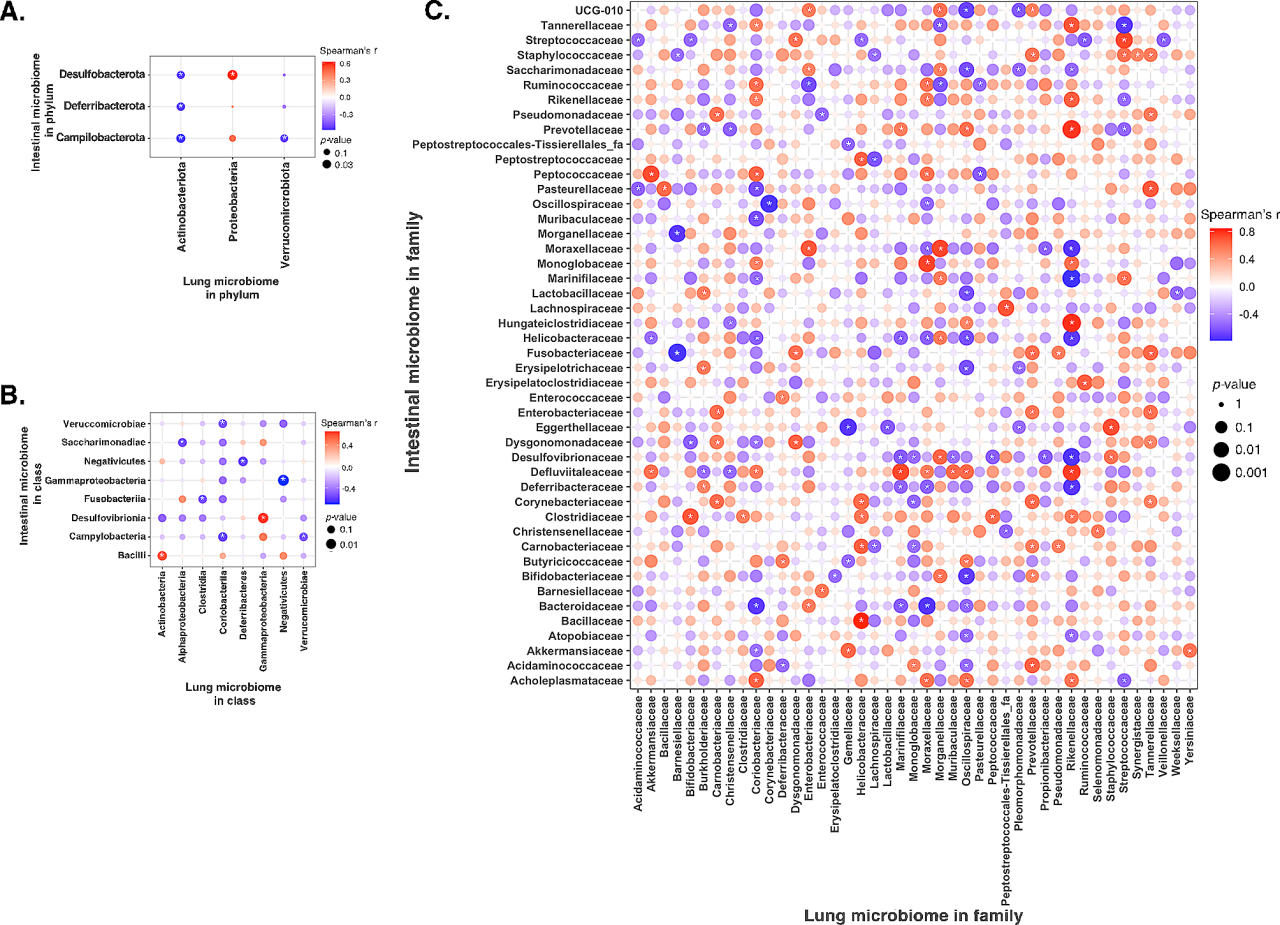
Heatmaps depicting correlation analyses. **(A)** Heatmap showing correlations between the lung and the intestinal microbiome at the phylum level. **(B)** Heatmap illustrating correlations between the lung and the intestinal microbiome at the class level. **(C)** Heatmap presenting correlations between the lung and the intestinal microbiome at the family level. The intensity of color indicates the correlation coefficient strength (red: positive correlation; blue: negative correlation). Size of the point reflects the significance of the correlation. * *p* < 0.05

Lung and gut microbiome correlation in phylum level showed that lung Actinobacteriota was negatively correlated with intestinal Campilobacterota, Defferibacterota, and Desulfobacterota (*p* < 0.05) (Fig. 7A). Lung Proteobacterota was positively correlated with intestinal Desulfobacterota whereas the lung Verrucomicrobiota was negatively correlated with intestinal Campilobacterota (*p* < 0.05). In the class level, lung Actinobacteria and Gammaproteobacteria were positively correlated with intestinal Bacilli and Desulfovibrionia (*p* < 0.05) (Fig. 7B). The lung Alphaproteobacteria, Clostridia, Coriobacteriia, Deferribacteres, Negativicutes, and Verrucomicrobiae were negatively correlated with intestinal Campylobacteria, Fusobacteria, Gammaproteobacteria, Negativicutes, Saccharimonadiae, and Verrucomicrobiae (*p* < 0.05). Further analysis in family level showed that 43 of the identified microbiome in lung were correlated with 46 of the microbiome in intestine (*p* < 0.05) (Fig. 7C). Of those, we identified positive correlations between 31 lung microbiome families and 41 intestinal microbiome families, and negative correlations between 32 lung microbiome families and 40 intestinal microbiome families (*p* < 0.05). Particularly, the Clostridiaceae, Dysgonomonadaceae, Rikenellaceae, and Streptococcaceae in the lung were positively correlated, whereas Moraxellaceae were negatively correlated with their counterparts in the intestine (*p* < 0.05).

### Correlation between intestinal microbiome and lung inflammation

We observed significant correlations between intestinal microbiome and lung inflammatory markers (Figure S4). In the intestinal phylum, Bacteroidota was positively correlated with IL-17 A, IFN-ɣ, and 8-isoprostane while Verrucomicrobiota was positively correlated with IFN-ɣ and 8-isoprostane. Detailed results were presented in section S2.2 in SI.

### Concordant intestinal microbiome dysbiosis in COPD GOLD stage 4 patients and CS-exposed mice

**Fig. 8:**
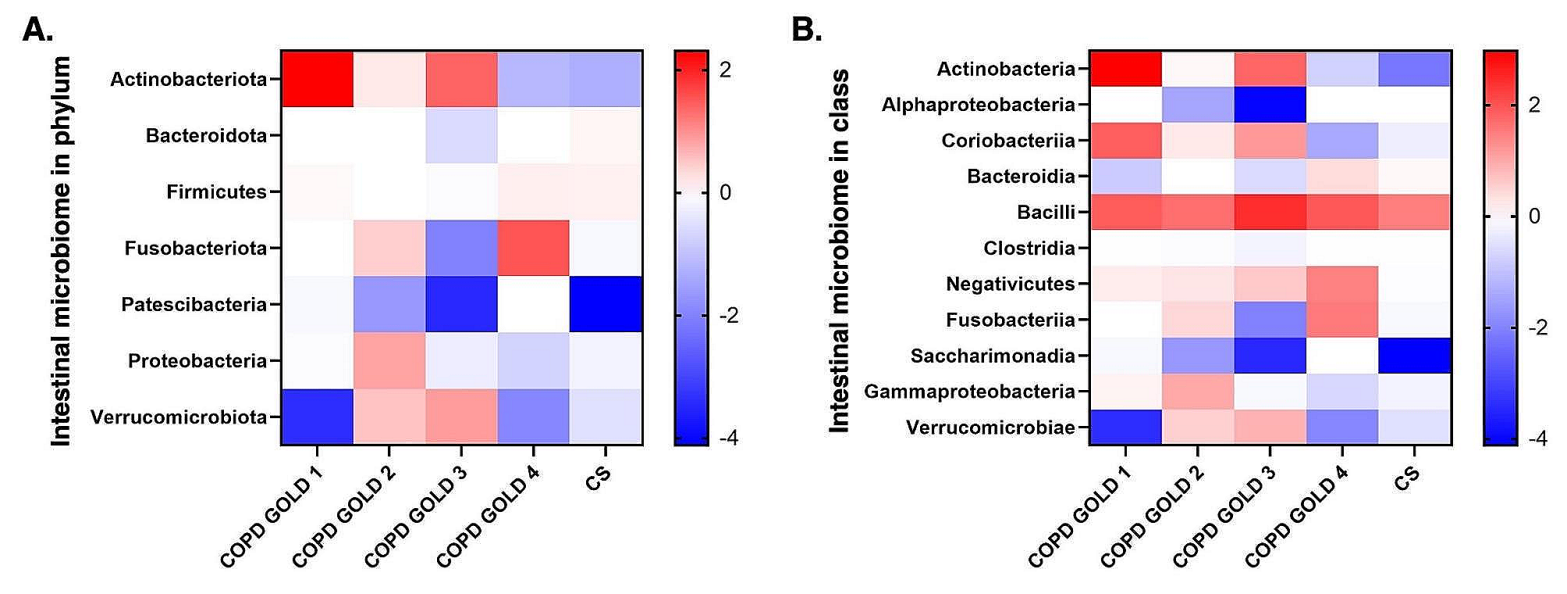
Hierarchical clustering heatmaps. Heatmaps showing relative abundance of intestinal microbiome in chronic obstructive pulmonary disease (COPD) of different Global Initiative for Chronic Obstructive Lung Disease (GOLD) stage subjects and cigarette smoke (CS)-exposed mice compared to the respective control **(A)** at phylum level and **(B)** at class level. The color depth represents the relative abundance of the microbiome, with red indicating increased relative abundance and blue indicating decreased relative abundance

We found decreases in Patescibacteria, Proteobacteria, and Verrucomicrobiota, with an increase in Firmicutes in COPD GOLD stage 1 patients consistent with the dysbiosis in CS-exposed mice (Fig. 8A). Similarly, in COPD GOLD stage 2 patients, a reduction in Patescibacteria mirrored findings from CS-exposed mice. In patients with COPD GOLD stage 3, reductions in Patescibacteria and Proteobacteria were found, mirroring findings in CS-exposed mice. Notably, patients of COPD GOLD stage 4 exhibited decreases in Actinobacteriota and increases in Firmicutes, aligning closely with microbiome changes seen in CS-exposed mice. In the class level, patients with COPD GOLD stage 1 exhibited decrease in Clostridia, Saccharimonadia, and Verrucomicrobiae, and increase in Bacilli, similar to those in CS-exposed mice (Fig. 8B). In patients with COPD GOLD stage 2, decreases in Clostridia and Saccharimonadia, coupled with an increase in Bacilli, mirrored the dysbiosis in CS-exposed mice. In COPD GOLD stage 3 patients, decrease in Clostridia, Fusobacteriia, Saccharimonadia, and Gammaproteobacteria, alongside an increase in Bacilli, paralleled the patterns seen in CS-exposed mice. Interestingly, individuals in COPD GOLD stage 4 showed high resemblance pattern with CS-exposed mice microbiome, with decreases in Actinobacteria, Alphaproteobacteria, Clostridia, Gammaproteobacteria, and Verrucomicrobiae, and increases in Bacteroidia and Bacilli.

## Discussion

This study’s significance lies in its investigation of the effects of CS on the lung and intestinal microbiomes in mice, a novel approach in understanding the interplay between smoking and microbiome-related health effects. The key novelty is the identification of increased inflammatory markers in the lungs, correlating with dysbiosis in both lung and intestinal microbiomes in CS-exposed mice. Our study found that CS exposure significantly reduced lung function and induced lung and intestinal microbiome dysbiosis, correlating with increased inflammation. This changes, consistent in both CS-induced COPD mice and COPD patients, involved an increase in Firmicutes and a decrease in Patescibacteria and Verrucomicrobiota. This study provides critical insights into how smoking influences the lung and gut microbiomes, potentially contributing to respiratory and systemic health challenges in COPD.

We found that CS exposure reduced FVC in mice, mirroring observations in COPD patients where decreased FVC is often associated with incomplete exhalation due to increased airway wall thickness in severe cases [19]. This increase in airway wall thickness observed in our CS-exposed mice, is consistent with CT scan findings in smokers, suggesting a combination of inflammation and remodeling, a phenomenon less pronounced in former smokers [3]. Furthermore, we noted increased MLI in CS-exposed mice indicating emphysematous changes, which supported previous associations on smoking duration and emphysema severity [20]. These changes, along with airway wall thickening, confirm our observations of reduced FVC, suggesting restrictive airflow limitation. We also noted significant lung damage in CS-exposed mice characterized by extensive alveolar damage. This aligns with previous study showing lung damage and inflammation caused by reactive oxygen species (ROS) in rats exposed to CS [21]. Additionally, CS exposure markedly increased IL-1β, IL-10, IFN-ɣ, and 8-isoprostane in the serum and KC levels in the lungs, consistent with findings in mice exposed to CS for a short duration [22]. In COPD, emphysema is the main pathological feature of COPD which the well-established pathomorphological indicator include MLI in vivo [23, 24]. Previous studies reported that there is no single animal model that can reproduce all of the characteristics of COPD in humans [25, 26]. For example, the variable that determines patient diagnosis, the airflow obstruction, is not always taken into account in the models. Animal models of COPD are generally focused on the development of emphysema, airway remodeling, and changes in related molecular mediators [25, 26]. Therefore, the main experiment focus is on the ability of repeated whole-body CS exposure to induce various features of COPD, including decreased FVC, increased airway wall thickness, emphysema development, and increased inflammation, as observed in our CS-exposed mice. A recent meta-analysis study also reported that the prevalence of GOLD-COPD was consistently higher in men than in women, underscoring the importance of understanding the pathomechanisms in these individuals [27]. However, our findings in male mice may not necessarily apply to females due to sex difference being a significant biological determinant in inflammatory reactions and consequently lung function changes. For example, a previous study in adult male, female, and ovariectomized mice exposed to CS for 6 months reported increased tissue resistance, respiratory resistance, and lower inspiratory capacity in females, indicating an increased risk of small airway disease compared to males [28]. Another study found that female tracheal epithelial cells demonstrated greater barrier function and higher ciliary function than males, with more disruption in females [29]. Despite these apparent sex differences, the male mice in our study are still important for understanding the exact mechanisms causing inflammation and lung function abnormalities in CS exposure. Notably, our study observed increased inflammatory cell infiltration in the intestines of CS-exposed mice, similar to previous research in guinea pigs, which found changes in the intestinal tissue following CS exposure [30]. Another study reported similar intestinal effects, including shortened colon length and mucosal damage in C57BL/6 mice exposed to CS [31]. Thus, our findings suggest that CS-induced COPD results in both lung and colonic inflammation.

In our lung microbiome, we found that Bacteroidota, Firmicutes, and Proteobacteria were the predominant microbiome, consistent with previous study investigating bronchial wash samples from COPD patients, non-COPD smokers, and healthy subjects [32]. Notably, CS exposure led to an increase in Actinobacteriota and Coriobacteriia, which aligns with a reported elevated Actinobacteria levels in lung samples of COPD patients [33]. However, Einarsson et al. (2016) reported no significant variation in Actinobacteria abundance across different groups, suggesting that these disparities might be from shifts in the relative abundance of commensal bacteria [32]. Our study also revealed that at the family level, CS exposure increased the prevalence of Rikenellaceae, Coriobacteriaceae, Moraxellaceae, and Marinicellaceae, while decreasing Streptococcaceae and Christensellaceae. This trend of airway microbiome dysbiosis, particularly in smokers with a history of ≥ 10 pack-years, emphasize the impact of smoking on airway microbiome composition [34]. These findings collectively support our findings of lung microbiome dysbiosis in CS-exposed mice and suggest potential correlations with host response changes.

We observed that CS exposure increased microbiome richness but decreased the alpha diversity and evenness in the intestinal microbiome, with significant compositional differences between the control and CS-exposed groups. This finding is in line with a previous study, which reported differences in intestinal microbiome diversity between never smokers, former smokers, and current smokers [35]. At the class level, our analysis showed a decrease in the abundance of Patescibacteria, Campilobacterota, Defferibacterota, Actinobacteriota, and Desulfobacterota following CS exposure. Additionally, at the family level, we observed dysbiosis characterized by an increase in Acholeplasmataceae and Prevotellaceae, and a decrease in Marinifilaceae and Helicobacteraceae. Notably, Acholeplasmataceae and Hungateiclostridiaceae were only present post-CS exposure, while Helicobacteraceae were absent. These observations align with previous study on CS-exposed mice, which identified changes in microbiome composition, including presence of OTU010 representing Lachnospiraceae [36]. CS exposure may create an environment conducive to the growth of certain microbes, potentially leading to an increase in opportunistic pathogens, a decrease in commensal bacteria, or the introduction of foreign pathogens [37]. Together, these suggest that CS exposure not only affects microbial communities in the lungs but also in distant organs like the intestine, potentially linking intestinal dysbiosis to decreased lung function.

Next, we identified correlations between lung function and changes in both lung and intestinal microbiomes in CS-exposed mice. Earlier studies have linked changes in the composition of the lung microbiome and compromised lung immunity to a decline in lung function [9, 38]. Similarly, intestinal microbiome dysbiosis has been associated with immunological shifts and lung diseases development [39]. Notably, intestinal microbiomes from COPD patients could exacerbate mucus hypersecretion in lung of mice, accelerating decreased lung function [40]. Our findings also revealed correlations between inflammatory markers in the lung and intestine and microbiome dysbiosis. This is in line with a study that reported microbiome diversity in bronchoalveolar lavage fluid from interstitial lung fibrosis patients was correlated with increased alveolar cytokines [41]. Furthermore, we discovered a correlation between intestinal microbiome dysbiosis and lung microbiome changes following CS exposure, with multiple of the same microbiome taxa in both the lung and the intestine were correlated. The intestinal microbiome was also correlated with lung inflammatory markers, underscoring the gut-lung axis’s bidirectional relationship. This axis highlights the interconnectedness of microbiomes in various organs and their influence on inflammatory responses, potentially through the mesenteric lymphatic system transporting bacteria and their by-products to the lungs [42]. These findings collectively support the notion that CS-induced dysbiosis in both lung and intestinal microbiomes contributes to lung alveolar damage and emphysema development.

We observed dysbiosis pattern in intestinal samples of COPD patients showing similar pattern as those in the intestines of CS-exposed mice in our study. Particularly, patients with COPD GOLD stage 4 showed high concordant microbiome dysbiosis with those of CS-exposed mice, indicating similarities in microbiome composition [10, 43]. Notably, the dominance of Firmicutes and Bacteroidetes in the human intestinal microbiome, as reported in previous studies, mirrors our observations in mice, further highlighting these similarities. A previous study comparing stool microbiome between different GOLD stages in COPD patients showed Bacteroidetes was more abundant in stage 1 COPD than stage 2–4 COPD while Fusobacterium and Aerococcus were more abundant in stage 3 and 4 [44]. Another study in COPD patients showed a 62% reduction in COPD incidence with the presence of Patescibacteria, corroborating our observation of decreased Patescibacteria in all GOLD stage of COPD patients and CS-exposed mice [43]. However, the authors also noted no distinct separation in microbiome communities between COPD and non-COPD subjects. This suggests that while the overall microbial community might not be directly associated with COPD incidence, specific microbial taxa changes, like those we identified, could be influential. These insights imply that the CS-exposed mouse model mirrors specific microbiome dysbiosis seen in COPD subjects. Therefore, this mice model could be useful in further exploring the links between microbiome dysbiosis and COPD in humans.

There are limitations in our work. The findings may benefit from larger sample sizes, and using both male and female mice in our future study may reveal more comprehensive interactions between the microbiome, inflammation, and lung changes. Additionally, the absence of direct clinical data from human subjects limits the direct translation of our findings to real-world scenarios despite our efforts in assessing the comparability of these changes with those observed in COPD. The number of publicly available databases of microbiome specimens from lung of COPD patients is also limited, with the majority consisting of sputum and upper airway samples and may be explored in future study to see its similarities to the mice lung microbiome. Future studies involving human subjects, incorporating smoking status and microbiome assessments, may be needed to enhance the generalizability of our results. Such findings will provide a better understanding of the impact of observed microbiome changes and lung impairments in human subjects.

## Conclusions

In summary, our study reveals that chronic CS exposure in mice causes dysbiosis in both lung and intestinal microbiomes, leading to decreased lung function, increased airway wall thickness, emphysema, and lung injury. This dysbiosis is also correlated with lung function changes and inflammation, with similar patterns observed in CS-exposed mice and COPD, particularly of GOLD stage 4, patients. These findings underscore the potential clinical implications of CS exposure in contributing to lung impairment through microbiome dysbiosis. Understanding these mechanisms could pave the way for novel therapeutic strategies targeting microbiome modulation, offering potential avenues for treating or preventing lung diseases associated with smoking.

### Electronic supplementary material

Below is the link to the electronic supplementary material.

Supplementary Material 1

## Data Availability

The datasets used and/or analysed during the current study are available from the corresponding author on reasonable request.

## Abbreviations

COPD: Chronic obstructive pulmonary disease
CS: Cigarette smoke
CXCL1/KC: Chemokine (CXC motif) ligand 1/keratinocyte chemoattractant
ELISA: Enzyme-linked immunosorbent assay
FEV0.1: Forced expiratory volume at 0.1 s
FVC: Forced vital capacity
GOLD: Global Initiative for Chronic Obstructive Lung Disease
H&E: Hematoxylin and eosin
HEPA: High efficiency particulate air
IFN: Interferon
IL: Interleukin
MLI: Mean linear intercept
PCoA: Principal coordinate analysis
PEF: Peak expiratory flow
rDNA: Ribosomal DNA
RH: Relative humidity
SI: Supplementary information
UniFrac: Unique fraction
