# Supplementary material for: Cigarette smoke-induced dysbiosis: comparative analysis of lung and intestinal microbiomes in COPD mice and patients
Source: Respir Res. 2024 May 10;25:204. doi: 10.1186/s12931-024-02836-9 (PMC11088139; doi:10.1186/s12931-024-02836-9)
Supplement: Supplementary file 1 — Supplementary Material 1 [file 12931_2024_2836_MOESM1_ESM.docx]

**Supplementary Information**

**Cigarette Smoke-Induced Dysbiosis: Comparative Analysis of Lung and Intestinal Microbiomes in COPD Mice and Patients**

Vincent Laiman^1#^, Hsiao-Chi Chuang^2,3,4,5#^, Yu-Chun Lo^6^, Tzu-Hsuen Yuan^7^, You-Yin Chen^6,8,9^, Didik Setyo Heriyanto^10^, Fara Silvia Yuliani^11^, Kian Fan Chung^5^, Jer-Hwa Chang^2,12*^

^1^Department of Radiology, Faculty of Medicine, Public Health, and Nursing, Universitas Gadjah Mada – Dr. Sardjito Hospital, Yogyakarta, Indonesia

^2^School of Respiratory Therapy, College of Medicine, Taipei Medical University, Taipei, Taiwan

^3^Division of Pulmonary Medicine, Department of Internal Medicine, Shuang Ho Hospital, Taipei Medical University, New Taipei City, Taiwan

^4^Cell Physiology and Molecular Image Research Center, Wan Fang Hospital, Taipei Medical University, Taipei, Taiwan

^5^National Heart and Lung Institute, Imperial College London, London, UK

^6^The Ph.D. Program for Neural Regenerative Medicine, College of Medical Science and Technology, Taipei Medical University, Taipei, Taiwan

^7^Department of Health and Welfare, College of City Management, University of Taipei, Taipei, Taiwan

^8^Industrial Ph.D. Program of Biomedical Science and Engineering, National Yang Ming Chiao Tung University, Taipei, Taiwan

^9^Department of Biomedical Engineering, National Yang Ming Chiao Tung University, Taipei, Taiwan

^10^Department of Anatomical Pathology, Faculty of Medicine, Public Health, and Nursing, Universitas Gadjah Mada – Dr. Sardjito Hospital, Yogyakarta, Indonesia

^11^Department of Pharmacology and Therapy, Faculty of Medicine, Public Health, and Nursing, Universitas Gadjah Mada, Yogyakarta, Indonesia

^12^Division of Pulmonary Medicine, Departments of Internal Medicine, Wan Fang Hospital, Taipei Medical University, Taipei, Taiwan

**^#^These authors contributed equally to this work.**

***Corresponding Author**

*Jer-Hwa Chang, MD., MSc.*

School of Respiratory Therapy, College of Medicine, Taipei Medical University, 250 Wuxing Street, Taipei 11031, Taiwan.

Telephone: +886-2-27361661. Fax: +886-2-27391143. E-mail: [m102094030@tmu.edu.tw](mailto:m102094030@tmu.edu.tw)

**Materials and methods**

*S1.1. Cigarette smoke (CS)-induced emphysema model*

The whole CS from the combustion of 32 commercial cigarettes (Longlife, Original, Taiwan) equivalent to 8 mg nicotine and 10 mg tar was introduced into the whole-body exposure chamber at a flow rate of 1.5 L/min. There were 16 slots of cigarettes tubes equipped with electronic lighting controls, and the cigarettes underwent continuous combustion without air suction. DustTrak monitor (8532, TSI, Shoreview, MN, USA) was used to measure the mass concentrations of particulate matter of ≤ 2.5 μm in aerodynamic diameter (PM_2.5_). The PM_2.5_ emitted from cigarette burning was measured 1-2 times (1 time/cigarette) daily. During cigarette burning, the PM_2.5_ reached the peak concentration of 37.8 mg/m^3^ in the first 4 mins of combustion and the lowest concentration of 18.43 mg/m^3^ at 8 mins of combustion (Figure S1B). The average PM_2.5_ mass concentration during the study period was 30.45±27.79 mg/m^3^. The mice were placed in normal air for one week after the exposure to mitigate the short-term impacts of CS exposure, resulting in a more accurate representation of the long-term effect of CS on the microbiome.

*S1.2. Microbiotic DNA preparation and analysis*

The QIAamp DNA Stool Mini Kit (Qiagen) were used to extract intestinal bacterial DNA following the manufacturers’ instructions. The minimum final concentration of intestinal and lung bacterial DNA samples was 5 ng/µL, and all DNA samples were stored at -80 °C. Universal 16S ribosomal (r)RNA gene primers V3 (341F, 5’-CCTACGGGNGGCWGCAG-3’) and V4 (805R, 5’-GACTACHVGGGTATCTAATCC-3’) were recommended and designed by Illumina (<https://support.illumina.com/downloads/16s_metagenomic_sequencing_library_preparation.html>). These two primers involved overhang adapter sequences in the forward (5’-TCGTCGGCAGCGTCAGATGTGTATAAGAGACAG-3') and reverse (5’-GTCTCGTGGGCTCGGAGATGTGTATAAGAGACAG-3') primers and amplified the targeted sequence of the bacterial 16S rRNA gene. In addition, a limited cycle polymerase chain reaction (PCR) amplified the V3-V4 region of the bacterial 16S rRNA gene to construct the amplicon library. The sequencing libraries, Illumina sequencing adapters, and dual-index barcodes were attached to the amplicon library. To ensure that the amount was sufficient to attach 16S rRNA, the quantity and quality of the sequencing libraries were confirmed by a QSep100 analyzer (BiOptic, New Taipei City, Taiwan). The v3 chemistry generated paired-end reads of 300 bases in length to normalize the libraries, pool the library in an equimolar ratio, and sequence them on Illumina Miseq.

After 16S rRNA sequencing, the universal primer sequence and low-quality reads were removed. The following process and analysis were executed with the phyloseq workflow of the DADA2 package (vers. 1.6) in *R* environment. Functions of the DADA2 package included filtering, trimming, de-replication, and de-noising of the forward and reverse reads. After merging the processed overlapping paired-end reads, chimers were removed from the cleaned full-length amplicons. Taxonomic assignment of the inferred amplicon sequence variants (ASVs) was performed using the SILVA reference database (vers. 132) with minimum bootstrap confidence of 80. Multiple sequences were aligned to ASVs with the DECIPHER package (vers. 2.6.0), and RAxML (vers. 8.2.11) was used to construct a phylogenetic tree. The phyloseq package (vers. 1.22.3) created a phyloseq object for downstream bacterial community analyses based on the frequency table, taxonomy, and phylogenetic tree information.

**Results**

*S2.1. Correlation between intestinal inflammation and intestinal microbiome*

Figure S2 shows the correlation between intestinal inflammation and intestinal microbiome. The IL-1β was positively correlated with Morganellaceae (*p*<0.05). The IL-10, IFN-ɣ, and KC were all positively correlated with Bacillaceae, Hungateiclostridiaceae, Morganellaceae, Peptostreptococcaceae, and Staphylococcaceae (*p*<0.05). Additionally, IL-10 was also positively correlated with Fusobacteriota, Fusobacteriia, Carnobacteriaceae, Corynebacteriaceae, and Fusobacteriaceae (*p*<0.05). Negative correlation was observed between IFN-ɣ and Marinifilaceae (*p*<0.05). The 8-isoprostane correlated positively with Acholeplasmataceae and Hungateiclostridiaceae while negatively with Campilobacterota, Campylobacteria, Coriobacteriaceae, Helicobacteraceae, and Moraxellaceae (*p*<0.05).

*S2.2. Correlation between intestinal microbiome and lung inflammation*

Figure S3 shows the correlation between intestinal inflammation and intestinal microbiome. The IL-1β was negatively correlated with Bacilli while the IL-10 was negatively correlated with Peptococcaceae (*p*<0.05). The IL-17A was positively correlated with Bacteroidota, Bacteroidia, and Muribaculaceae while negatively correlated with Lachnospiraceae and Ruminococcaceae (*p*<0.05). The IFN-ɣ was positively correlated with Bacteroidota, Verrucomicrobiota, Bacteroidia, Verrucomicrobiae, Akkermansiaceae, Muribaculaceae, and Staphylococcaceae while negatively correlated with Firmicutes, Peptococcaceae, Rikenellaceae, Ruminococcaceae, and Tannerellaceae (*p*<0.05). The KC was negatively correlated with Actinobacteriota, Desulfobacterota, Patescibacteria, Actinobacteria, Desulfovibrinia, Saccharimonadia, Bifidobacteriaceae, Desulfovibrionaceae, and Saccharimonadaceae while positively correlated with Tannerellaceae. The 8-isoprostane was positively correlated with Bacteroidota, Verrucomicrobiota, Bacteroidia, Verrucomicrobiae, Akkermansiaceae, Muribaculaceae, Peptostreptococcaceae, and Staphylococcaceae (*p*<0.05).

**Figure S1.** Schematic experimental design of cigarette smoke (CS)-exposed mice model. **(A)** Mice were exposed to CS for 20 weeks using a CS generation system following equipped with a whole-body exposure system for 8 hours a day, 5 days a week. Mice were then sacrificed at week 21. **(B)** Distribution of particulate matter of ≤ 2.5 µm in aerodynamic diameter (PM_2.5_) in mass concentrations during a cigarette burning.

**
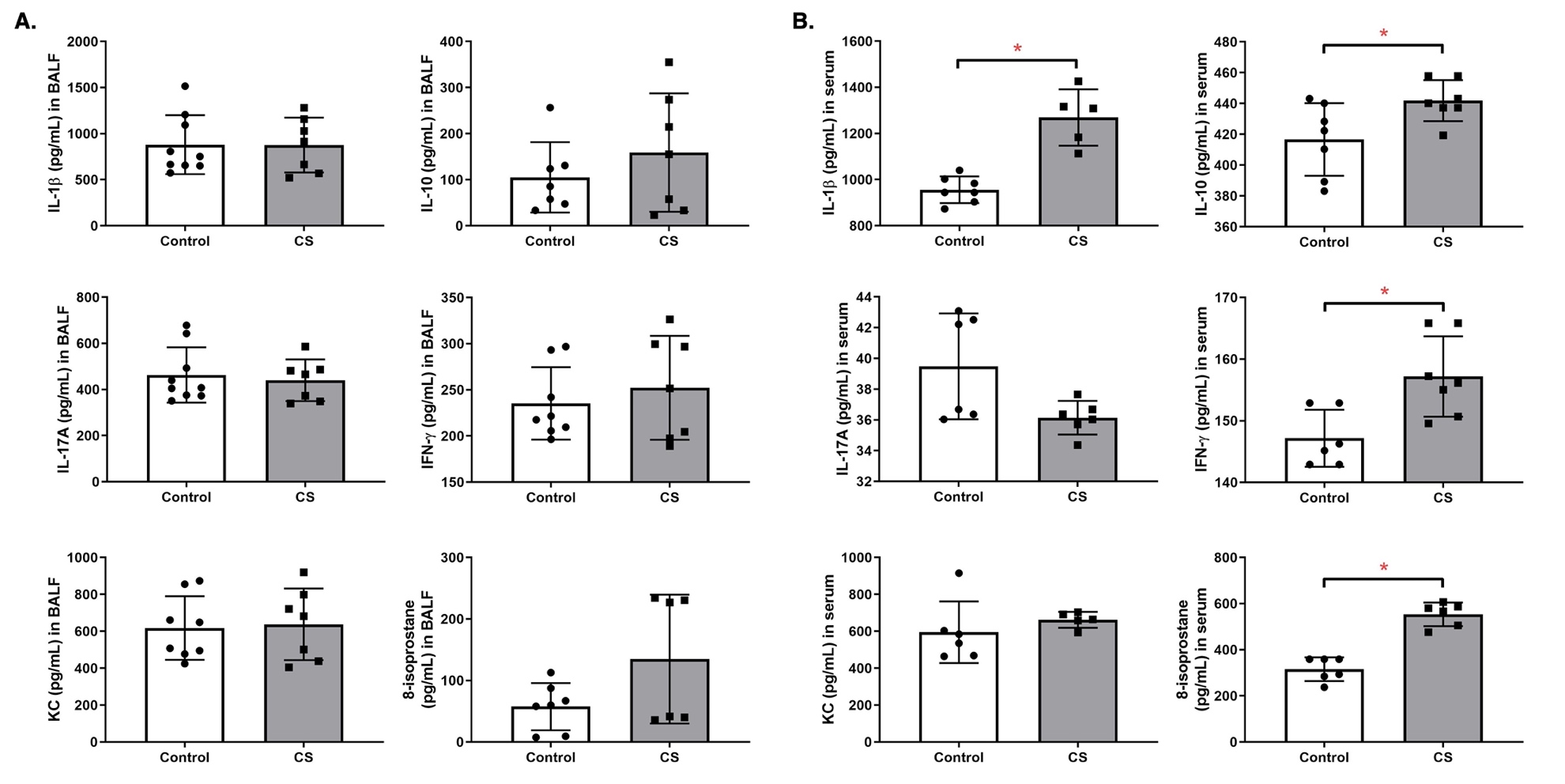
**

**Figure S2.** Inflammatory markers including interleukin (IL)-1β, IL-10, IL-17A, interferon (IFN)-ɣ, keratinocyte chemoattractant (KC), and 8-isoprostane in **(A)** bronchoalveolar lavage (BALF) (*n* = 6-9) and **(B)** serum (*n* = 5-7). ^*^ *p* < 0.05.

**Figure S3.** Correlation analysis heatmap between the intestinal microbiome and the intestinal inflammatory markers. The depth of the color indicates strength of correlation coefficient (red: positive correlation; blue: negative correlation). Size of the point indicates the correlation significance. * *p*<0.05


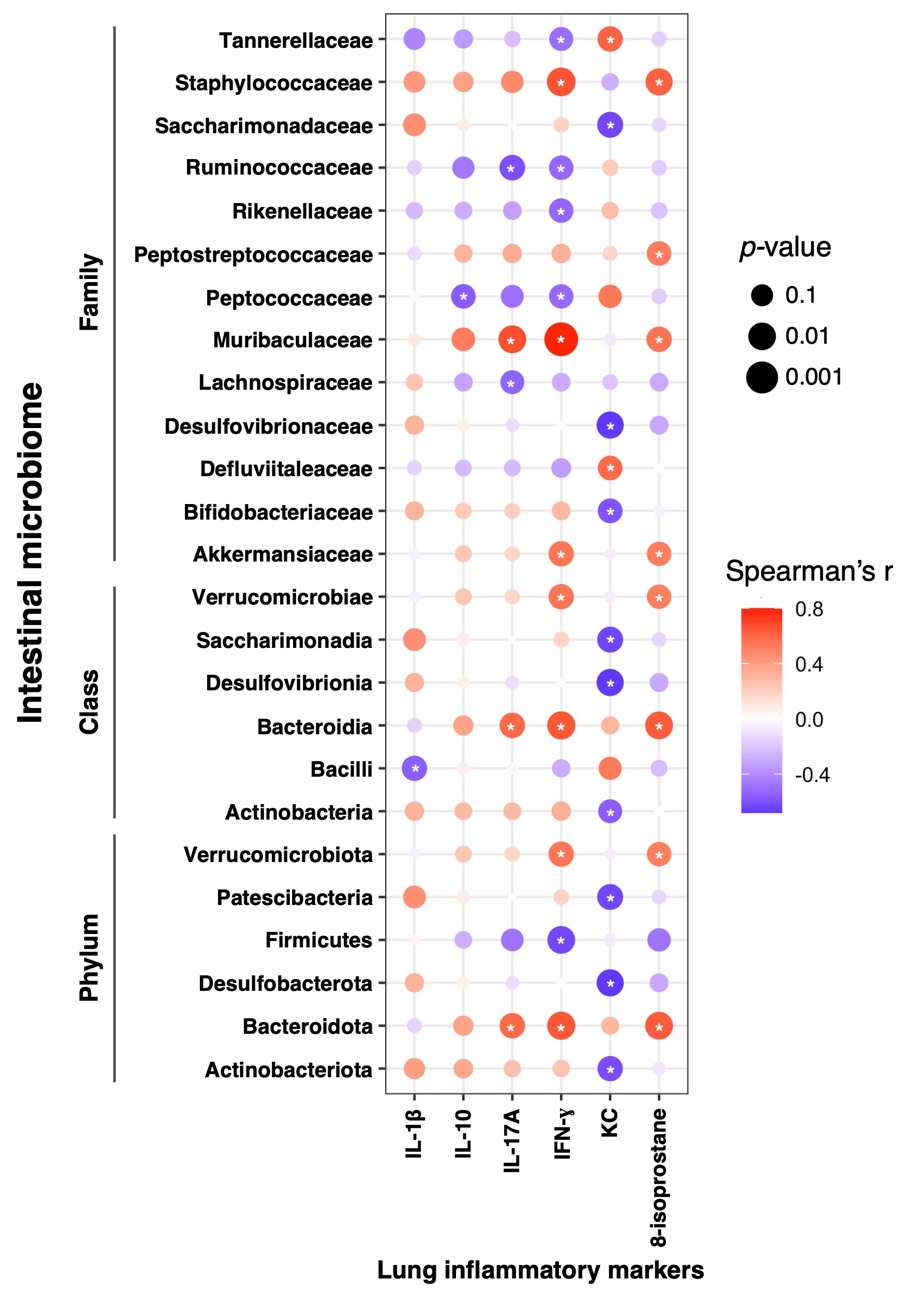


**Figure S4.** Correlation analysis heatmap between the intestinal microbiome and the lung inflammatory markers. The depth of the color indicates strength of correlation coefficient (red: positive correlation; blue: negative correlation). Size of the point indicates the correlation significance. * *p*<0.05
